# 2-(Biphenyl-4-yl)acetic acid (felbinac)

**DOI:** 10.1107/S1600536810035828

**Published:** 2010-09-25

**Authors:** Bernard Van Eerdenbrugh, Phillip E. Fanwick, Lynne S. Taylor

**Affiliations:** a Department of Industrial and Physical Pharmacy, Purdue University, 575 Stadium Mall Drive, West Lafayette, IN 47907, USA; b Laboratory for Pharmacotechnology and Biopharmacy, K.U. Leuven, Gasthuisberg O&N2, Herestraat 49, Box 921, 3000 Leuven, Belgium; c Department of Chemistry, Purdue University, 560 Oval Drive, West Lafayette, IN 47907, USA

## Abstract

The structure of the title compound, C_14_H_12_O_2_, displays the expected inter­molecular hydrogen bonding of the carb­oxy­lic acid groups, forming dimers. The dihedral angle between the two aromatic rings is 27.01 (7)°.

## Related literature

The title compound is a potent non-steroidal anti-inflammatory agent, used to treat muscle inflammation and arthritis. For single-crystal structures of inclusion complexes between felbinac and both hepta­kis-(2,3,6-tri-*O*-meth­yl)-β-cyclo­dextrin and β-cyclo­dextrin, see: Harata *et al.* (1992 ▶) and Wang *et al.* (2009 ▶), respectively. For single crystal structures of different complexes of felbinac with tryptamine and 1,2-diphenyl­ethyl­enediamine (different solvates), see: Koshima *et al.* (1998 ▶) and Imai *et al.* (2007 ▶), respectively.
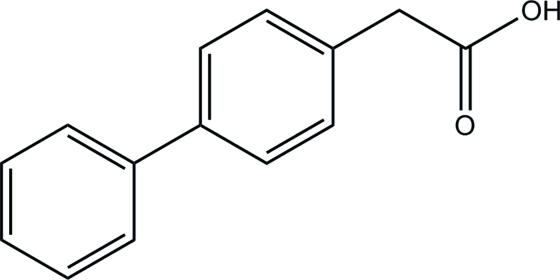


## Experimental

### 

#### Crystal data

C_14_H_12_O_2_
*M* *_r_* = 212.25
Orthorhombic, 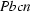

*a* = 46.248 (19) Å
*b* = 6.465 (3) Å
*c* = 7.470 (3) Å
*V* = 2233.4 (16) Å^3^
*Z* = 8
Cu *K*α radiation
μ = 0.64 mm^−1^
*T* = 150 K
0.20 × 0.20 × 0.20 mm

#### Data collection

Rigaku RAPID II diffractometer
Absorption correction: multi-scan (*CrystalClear*; Rigaku, 2001 ▶) *T*_min_ = 0.803, *T*_max_ = 0.881
8644 measured reflections
1952 independent reflections
1539 reflections with *I* > 2σ(*I*)
*R*_int_ = 0.037

#### Refinement

*R*[*F* ^2^ > 2σ(*F* ^2^)] = 0.038
*wR*(*F* ^2^) = 0.107
*S* = 1.08
1952 reflections
150 parameters
H atoms treated by a mixture of independent and constrained refinement
Δρ_max_ = 0.21 e Å^−3^
Δρ_min_ = −0.12 e Å^−3^

### 

Data collection: *CrystalClear* (Rigaku, 2001 ▶); cell refinement: *CrystalClear*; data reduction: *CrystalClear*; program(s) used to solve structure: *SIR2004* (Burla *et al.*, 2005 ▶); program(s) used to refine structure: *SHELXL97* (Sheldrick, 2008 ▶); molecular graphics: *ORTEPII* (Johnson, 1976 ▶) and *PLATON* (Spek, 2009 ▶); software used to prepare material for publication: *SHELXL97* and local programs.

## Supplementary Material

Crystal structure: contains datablocks global, I. DOI: 10.1107/S1600536810035828/vm2040sup1.cif

Structure factors: contains datablocks I. DOI: 10.1107/S1600536810035828/vm2040Isup2.hkl

Additional supplementary materials:  crystallographic information; 3D view; checkCIF report

## Figures and Tables

**Table 1:** Hydrogen-bond geometry (Å, °)

*D*—H⋯*A*	*D*—H	H⋯*A*	*D*⋯*A*	*D*—H⋯*A*
O2—H2⋯O1^i^	0.98 (2)	1.69 (2)	2.6663 (16)	178 (2)

Symmetry code: (i) 
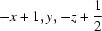
.

**Fig. 1.**
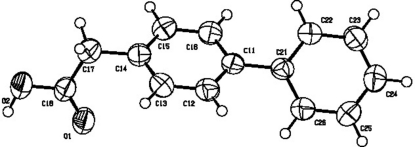
The molecular structure of the title compound, showing 50% probability displacement ellipsoids and the atomic numbering. H atoms are presented as small spheres of arbitrary radius.

## supplementary crystallographic information

### Comment

The title compound is a potent non-steroidal anti-inflammatory agent, used to treat muscle inflammation and arthritis. Although the single-crystal structures of inclusion complexes between felbinac and both heptakis-(2,3,6-tri-*O*-methyl)-β-cyclodextrin and β-cyclodextrin have been published (Harata *et al.*, 1992; Wang *et al.*, 2009), that of the pure compound has not been reported. The molecular structure is shown in Figure 1. The expected H-bonded carboxylic acid dimers are formed, with O1···O2 distances of 2.6663 (13) Å. The dihedral angle between the two benzene rings is 27.01 (7)°. Hydrogen bonds between carboxylic acid groups of felbinac are disrupted in the published felbinac-cyclodextrin structures (Harata *et al.*, 1992; Wang *et al.*, 2009). In the inclusion complex between felbinac and heptakis-(2,3,6-tri-*O*-methyl)-β-cyclodextrin (Harata *et al.*, 1992), no dimers are formed; in that between felbinac and β-cyclodextrin (Wang *et al.*, 2009), face-to-face π-π stackings form the basis for dimer formation. Hydrogen bonds between carboxylic acid groups of felbinac are disrupted in the complexes with tryptamine (Koshima *et al.*, 1998) and 1,2-diphenylethylenediamine (Imai *et al.*, 2007) due to ionic interactions with the amine functions.

### Experimental

A solution of 2-(biphenyl-4-yl)acetic acid (15 mg ml^-1^) was prepared in diethylether. Subsequently, 15 ml of the solution was transferred into a clean crystallization dish (diameter 50 mm; height 35 mm). The vessel was partially covered with a plastic sheet and the solution was allowed to slowly evaporate overnight.

### Refinement

The H atom bound to oxygen O2 was located in a difference Fourier map and refined freely with isotropic displacement parameters. Other H atoms were placed in calculated positions and treated as riding on their parent atoms with C—H = 0.95 Å (aromatic), 0.99 Å (aliphatic) and with Uiso(H) = 1.2Ueq(C).

### Crystal data

**Table:**

C_14_H_12_O_2_	*F*(000) = 896
*M**_r_* = 212.25	*D*_x_ = 1.262 Mg m^−^^3^
Orthorhombic, *P**b**c**n*	Cu - *K*α radiation, λ = 1.54184 Å
Hall symbol: -P 2n 2ab	Cell parameters from 8644 reflections
*a* = 46.248 (19) Å	θ = 6–66°
*b* = 6.465 (3) Å	µ = 0.64 mm^−^^1^
*c* = 7.470 (3) Å	*T* = 150 K
*V* = 2233.4 (16) Å^3^	Chunk, colourless
*Z* = 8	0.20 × 0.20 × 0.20 mm

### Data collection

**Table:**

Rigaku RAPID II diffractometer	1539 reflections with *I* > 2σ(*I*)
confocal optics	*R*_int_ = 0.037
ω scans	θ_max_ = 66.5°, θ_min_ = 6.6°
Absorption correction: multi-scan (*CrystalClear*; Rigaku, 2001)	*h* = −53→54
*T*_min_ = 0.803, *T*_max_ = 0.881	*k* = −7→7
8644 measured reflections	*l* = −8→8
1952 independent reflections	

### Refinement

**Table:**

Refinement on *F*^2^	H atoms treated by a mixture of independent and constrained refinement
Least-squares matrix: full	*w* = 1/[σ^2^(*F*_o_^2^) + (0.0586*P*)^2^ + 0.0731*P*] where *P* = (*F*_o_^2^ + 2*F*_c_^2^)/3
*R*[*F*^2^ > 2σ(*F*^2^)] = 0.038	(Δ/σ)_max_ = 0.001
*wR*(*F*^2^) = 0.107	Δρ_max_ = 0.21 e Å^−^^3^
*S* = 1.08	Δρ_min_ = −0.12 e Å^−^^3^
1952 reflections	Extinction correction: *SHELXL97* (Sheldrick, 2008)
150 parameters	Extinction coefficient: 0.92E-02
0 restraints	

### Special details

**Table:**

Geometry. All e.s.d.'s (except the e.s.d. in the dihedral angle between two l.s. planes) are estimated using the full covariance matrix. The cell e.s.d.'s are taken into account individually in the estimation of e.s.d.'s in distances, angles and torsion angles; correlations between e.s.d.'s in cell parameters are only used when they are defined by crystal symmetry. An approximate (isotropic) treatment of cell e.s.d.'s is used for estimating e.s.d.'s involving l.s. planes.
Refinement. Outlier data were removed using a local program based on the method of Prince and Nicholson.Refinement on *F*^2^ for ALL reflections except for 0 with very negative *F*^2^ or flagged by the user for potential systematic errors. Weighted *R*-factors *wR* and all goodnesses of fit *S* are based on *F*^2^, conventional *R*-factors *R* are based on *F*, with *F* set to zero for negative *F*^2^. The observed criterion of *F*^2^ > σ(*F*^2^) is used only for calculating R_factor_obs *etc*. and is not relevant to the choice of reflections for refinement. *R*-factors based on *F*^2^ are statistically about twice as large as those based on *F*, and *R*- factors based on ALL data will be even larger.

### Fractional atomic coordinates and isotropic or equivalent isotropic displacement parameters (Å^2^)

**Table:**

	*x*	*y*	*z*	*U*_iso_*/*U*_eq_	
O1	0.465961 (19)	0.26139 (14)	0.29970 (13)	0.0659 (3)	
O2	0.50183 (2)	0.25189 (14)	0.49623 (15)	0.0673 (3)	
C11	0.36381 (3)	0.24813 (16)	0.46896 (16)	0.0459 (3)	
C12	0.38092 (3)	0.07334 (19)	0.44073 (17)	0.0537 (3)	
C13	0.40981 (3)	0.0723 (2)	0.48829 (18)	0.0580 (4)	
C14	0.42282 (3)	0.24426 (18)	0.56461 (17)	0.0519 (4)	
C15	0.40583 (3)	0.4174 (2)	0.59492 (18)	0.0571 (4)	
C16	0.37693 (3)	0.41946 (19)	0.54734 (17)	0.0549 (4)	
C17	0.45452 (3)	0.24152 (19)	0.61233 (19)	0.0606 (4)	
C18	0.47426 (3)	0.25310 (17)	0.45355 (19)	0.0513 (4)	
C21	0.33265 (3)	0.25021 (16)	0.41626 (16)	0.0462 (3)	
C22	0.31270 (2)	0.37583 (18)	0.50399 (17)	0.0543 (4)	
C23	0.28391 (3)	0.3774 (2)	0.45408 (18)	0.0591 (4)	
C24	0.27427 (3)	0.25357 (19)	0.3176 (2)	0.0594 (4)	
C25	0.29358 (3)	0.1274 (2)	0.2301 (2)	0.0628 (4)	
C26	0.32239 (3)	0.1263 (2)	0.27863 (18)	0.0566 (4)	
H2	0.5133 (4)	0.258 (2)	0.386 (3)	0.105 (7)*	
H12	0.3726	−0.0465	0.3881	0.064*	
H13	0.4210	−0.0489	0.4683	0.070*	
H15	0.4141	0.5363	0.6491	0.069*	
H16	0.3658	0.5404	0.5686	0.066*	
H22	0.3190	0.4617	0.5996	0.065*	
H23	0.2707	0.4652	0.5149	0.071*	
H24	0.2545	0.2549	0.2839	0.071*	
H25	0.2871	0.0405	0.1357	0.075*	
H26	0.3355	0.0388	0.2164	0.068*	
H17A	0.4587	0.3598	0.6925	0.073*	
H17B	0.4588	0.1130	0.6794	0.073*	

### Atomic displacement parameters (Å^2^)

**Table:**

	*U*^11^	*U*^22^	*U*^33^	*U*^12^	*U*^13^	*U*^23^
O1	0.0465 (6)	0.0959 (8)	0.0552 (6)	−0.0007 (4)	−0.0058 (4)	0.0046 (5)
O2	0.0459 (6)	0.0945 (9)	0.0617 (6)	0.0005 (4)	−0.0098 (5)	−0.0014 (5)
C11	0.0472 (7)	0.0521 (8)	0.0384 (7)	−0.0008 (5)	0.0040 (5)	−0.0001 (5)
C12	0.0536 (8)	0.0521 (7)	0.0555 (8)	−0.0006 (6)	0.0023 (6)	−0.0078 (6)
C13	0.0545 (8)	0.0584 (8)	0.0611 (9)	0.0074 (6)	0.0030 (6)	−0.0049 (7)
C14	0.0499 (8)	0.0642 (9)	0.0416 (7)	−0.0003 (5)	0.0006 (5)	0.0029 (6)
C15	0.0571 (8)	0.0584 (8)	0.0559 (8)	−0.0041 (6)	−0.0027 (6)	−0.0090 (7)
C16	0.0529 (8)	0.0530 (7)	0.0587 (8)	0.0032 (6)	−0.0010 (6)	−0.0090 (6)
C17	0.0550 (9)	0.0761 (10)	0.0508 (8)	0.0019 (6)	−0.0054 (6)	−0.0001 (7)
C18	0.0469 (7)	0.0527 (8)	0.0543 (8)	0.0006 (5)	−0.0104 (6)	−0.0015 (6)
C21	0.0485 (8)	0.0490 (7)	0.0411 (7)	−0.0024 (5)	0.0041 (5)	0.0017 (5)
C22	0.0539 (8)	0.0611 (9)	0.0479 (7)	0.0028 (6)	0.0003 (6)	−0.0072 (6)
C23	0.0518 (8)	0.0678 (9)	0.0576 (9)	0.0068 (6)	0.0047 (6)	−0.0003 (7)
C24	0.0487 (8)	0.0666 (9)	0.0628 (9)	−0.0047 (6)	−0.0040 (6)	0.0066 (7)
C25	0.0585 (9)	0.0688 (10)	0.0612 (9)	−0.0073 (6)	−0.0072 (6)	−0.0109 (7)
C26	0.0551 (8)	0.0603 (9)	0.0544 (8)	−0.0010 (6)	0.0025 (6)	−0.0103 (6)

### Geometric parameters (Å, °)

**Table:**

O1—C18	1.2128 (17)	C17—C18	1.499 (2)
O2—C18	1.3144 (15)	C17—H17A	0.9900
O2—H2	0.98 (2)	C17—H17B	0.9900
C11—C16	1.3921 (16)	C21—C26	1.3869 (17)
C11—C12	1.3957 (16)	C21—C22	1.3930 (16)
C11—C21	1.4937 (18)	C22—C23	1.3827 (16)
C12—C13	1.3822 (16)	C22—H22	0.9500
C12—H12	0.9500	C23—C24	1.3710 (18)
C13—C14	1.3870 (17)	C23—H23	0.9500
C13—H13	0.9500	C24—C25	1.3749 (18)
C14—C15	1.3865 (16)	C24—H24	0.9500
C14—C17	1.5088 (18)	C25—C26	1.3811 (16)
C15—C16	1.3830 (16)	C25—H25	0.9500
C15—H15	0.9500	C26—H26	0.9500
C16—H16	0.9500		
			
C18—O2—H2	108.7 (11)	C14—C17—H17B	108.80
C16—C11—C12	117.42 (12)	H17A—C17—H17B	107.70
C16—C11—C21	121.62 (10)	O1—C18—O2	122.48 (13)
C12—C11—C21	120.97 (11)	O1—C18—C17	124.01 (12)
C13—C12—C11	120.89 (12)	O2—C18—C17	113.50 (13)
C13—C12—H12	119.60	C26—C21—C22	117.31 (12)
C11—C12—H12	119.60	C26—C21—C11	121.35 (10)
C12—C13—C14	121.41 (11)	C22—C21—C11	121.34 (11)
C12—C13—H13	119.30	C23—C22—C21	121.02 (12)
C14—C13—H13	119.30	C23—C22—H22	119.50
C15—C14—C13	117.92 (12)	C21—C22—H22	119.50
C15—C14—C17	121.45 (11)	C24—C23—C22	120.62 (12)
C13—C14—C17	120.63 (11)	C24—C23—H23	119.70
C16—C15—C14	120.90 (12)	C22—C23—H23	119.70
C16—C15—H15	119.50	C23—C24—C25	119.27 (13)
C14—C15—H15	119.50	C23—C24—H24	120.40
C15—C16—C11	121.46 (11)	C25—C24—H24	120.40
C15—C16—H16	119.30	C24—C25—C26	120.31 (13)
C11—C16—H16	119.30	C24—C25—H25	119.80
C18—C17—C14	113.85 (12)	C26—C25—H25	119.80
C18—C17—H17A	108.80	C25—C26—C21	121.47 (12)
C14—C17—H17A	108.80	C25—C26—H26	119.30
C18—C17—H17B	108.80	C21—C26—H26	119.30
			
C16—C11—C12—C13	−0.35 (19)	C14—C17—C18—O2	179.61 (9)
C21—C11—C12—C13	179.48 (11)	C16—C11—C21—C26	152.94 (12)
C11—C12—C13—C14	−0.3 (2)	C12—C11—C21—C26	−26.89 (17)
C12—C13—C14—C15	1.1 (2)	C16—C11—C21—C22	−27.41 (17)
C12—C13—C14—C17	−178.81 (12)	C12—C11—C21—C22	152.77 (12)
C13—C14—C15—C16	−1.2 (2)	C26—C21—C22—C23	−0.55 (17)
C17—C14—C15—C16	178.71 (12)	C11—C21—C22—C23	179.79 (11)
C14—C15—C16—C11	0.5 (2)	C21—C22—C23—C24	0.58 (19)
C12—C11—C16—C15	0.25 (19)	C22—C23—C24—C25	−0.14 (19)
C21—C11—C16—C15	−179.59 (11)	C23—C24—C25—C26	−0.3 (2)
C15—C14—C17—C18	−106.63 (14)	C24—C25—C26—C21	0.3 (2)
C13—C14—C17—C18	73.25 (15)	C22—C21—C26—C25	0.09 (18)
C14—C17—C18—O1	−0.88 (18)	C11—C21—C26—C25	179.76 (12)

### Hydrogen-bond geometry (Å, °)

**Table:**

*D*—H···*A*	*D*—H	H···*A*	*D*···*A*	*D*—H···*A*
O2—H2···O1^i^	0.98 (2)	1.69 (2)	2.6663 (16)	178 (2)

Symmetry codes: (i) −*x*+1, *y*, −*z*+1/2.
